# Optical Coherence Tomography Biomarkers Predicting Progression to Atrophy in Non-Exudative Age-Related Macular Degeneration

**DOI:** 10.3390/diagnostics16101555

**Published:** 2026-05-20

**Authors:** Aleksandra Prus-Ludwig, Adam Wylęgała, Edward Wylęgała, Magdalena Kijonka, Bogumił Wowra

**Affiliations:** 1 Department of Ophthalmology, Railway Hospital in Katowice, Medical University of Silesia, 40-760 Katowice, Poland; 2 Experimental Ophthalmology Unit, Department of Biophysics, Railway Hospital in Katowice, Medical University of Silesia, 40-760 Katowice, Poland; 3 Chair and Clinical Department of Ophthalmology, Faculty of Medical Sciences in Zabrze, Medical University of Silesia, 40-760 Katowice, Poland

**Keywords:** OCT, progression biomarkers, non-exudative (dry) AMD, iRORA, cRORA, drusen, geographic atrophy

## Abstract

Age-related macular degeneration (AMD) is a leading cause of irreversible vision loss worldwide. Geographic atrophy (GA) is an advanced, currently incurable stage of non-exudative AMD and is characterized by progressive atrophy of the retinal pigment epithelium and outer retina, resulting in substantial visual impairment. Optical coherence tomography (OCT) has revolutionized the diagnosis and monitoring of AMD by enabling in vivo visualization of retinal microstructure and identification of imaging biomarkers associated with progression to late-stage disease. Improved understanding of these lesions may clarify disease pathogenesis and inform the development of new therapeutic strategies and clinical trial endpoints. This review summarizes OCT-based biomarkers reported as predictors of progression to late atrophic forms of AMD, with emphasis on early atrophic changes that precede GA.

## 1. Introduction

Age-related macular degeneration (AMD) is a chronic, progressive disorder of the macula that can lead to central vision loss. It is among the most common causes of irreversible blindness in older adults worldwide. By 2050, vision loss due to AMD is projected to affect more than 9 million people, with a higher burden in women [1]. AMD has a multifactorial pathogenesis involving aging, genetic susceptibility, environmental exposures, and molecular pathways [2,3,4]. The disease may progress from early and intermediate non-exudative stages to advanced forms, including geographic atrophy (GA; advanced non-exudative AMD) and macular neovascularization (exudative or “wet” AMD). Characteristic pathological processes include chronic inflammation, oxidative stress, and accumulation of extracellular lipid-rich deposits (drusen) between the retinal pigment epithelium (RPE) and Bruch’s membrane. These changes can impair transport of metabolites and nutrients between the choriocapillaris and photoreceptors, contributing to photoreceptor dysfunction and atrophy. Thickening of Bruch’s membrane and other age-related alterations may also promote VEGF-driven neovascular complications in susceptible eyes [5]. Advances in retinal imaging have transformed AMD diagnosis and monitoring, enabling precise visualization of structural changes and improved linkage of imaging findings to disease mechanisms.

Currently, several imaging modalities are used to diagnose non-exudative AMD. Core techniques for diagnosis and monitoring include color fundus photography (CFP), optical coherence tomography (OCT), fundus autofluorescence (FAF; blue–green), and near-infrared (NIR) imaging. Fluorescein angiography (FA), indocyanine green angiography (ICG-A), and OCT angiography (OCT-A) may also be used in selected cases. However, OCT is generally the preferred modality for structural assessment and longitudinal monitoring in AMD [6,7].

This review summarizes OCT biomarkers associated with AMD progression that are most consistently reported and clinically relevant based on their frequency and correlations in the literature. We focus on non-exudative AMD, particularly early atrophic changes that occur before the development of GA. Identifying higher-risk subgroups—especially among patients with intermediate AMD (iAMD)—is important for patient counseling, follow-up planning, and selection of candidates for future interventional studies aimed at preventing or delaying progression to GA.

## 2. The Importance of OCT in Non-Exudative AMD Imaging

Recent advances in noninvasive retinal imaging have improved the ability to quantitatively and qualitatively assess pathological features of non-exudative AMD. OCT has become a key modality for AMD evaluation because it provides cross-sectional and en face views of the retina, allowing detailed assessment of retinal layer architecture. High-resolution OCT enables precise analysis of structural alterations and supports identification of biomarkers associated with disease progression, which is important for staging and for developing future therapeutic strategies and trial endpoints [8,9].

**Figure 1:**
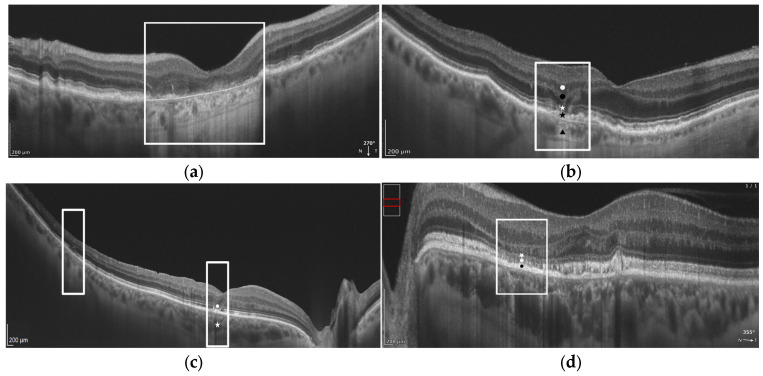
REVO high-resolution (HR) OCT B-scan presenting the four terms for atrophy defined by the CAM: (**a**) cRORA, including (the contents of the white rectangle) a region of choroidal hypertransmission with a diameter of at least 250 µm; a corresponding zone of RPE attenuation/disruption; and evidence of overlying photoreceptor degeneration (for details, please refer to the main text). (**b**) iRORA consisting of choroidal signal hypertransmission (black triangle) with a corresponding zone of RPE attenuation/disruption less than 250 µm (black star) and signs of overlying photoreceptor degeneration (e.g., subsidence of the inner nuclear layer (INL) (white dot) and outer plexiform layer (OPL) (black dot); a hyporeflective wedge in the Henle fiber layer (HFL); ONL thinning; and disruption of the ELM or EZ (white star)). (**c**) cORA defined by the absence of the EZ and IZ (white dot), marked thinning of the outer retina, and intermittent choroidal hypertransmission (white star), with an intact RPE band. (**d**) iORA characterized by a continuous ELM (white star) and a detectable but interrupted EZ (white dot) in the setting of noticeable outer retinal thinning, without choroidal hypertransmission and with an intact RPE band (black dot).

The importance of OCT in diagnosing AMD is demonstrated by the fact that the Classification of Atrophy Meetings (CAM) program established OCT-based criteria for diagnosing retinal atrophy at various stages of the disease [10,11]. Specifically, the following terms for the OCT findings were introduced:

- Complete retinal pigment epithelium (RPE) and outer retinal atrophy (cRORA): defined by (1) a region of choroidal hypertransmission at least 250 µm in diameter; (2) a corresponding zone of RPE attenuation/disruption at least 250 µm in diameter; and (3) evidence of overlying photoreceptor degeneration (e.g., outer nuclear layer (ONL) thinning, external limiting membrane (ELM) loss, and ellipsoid zone (EZ) and/or interdigitation zone (IZ) loss), in the absence of an RPE tear.
- Incomplete RPE and outer retinal atrophy (iRORA): characterized by choroidal signal hypertransmission with a corresponding zone of RPE attenuation/disruption and signs of overlying photoreceptor degeneration (e.g., subsidence of the inner nuclear layer (INL) and outer plexiform layer (OPL); a hyporeflective wedge in the Henle fiber layer (HFL); ONL thinning; and disruption of the ELM or EZ); the changes are insufficient to meet the definition of cRORA, and there is no evidence of an RPE tear.
- Complete outer retinal atrophy (cORA): defined by the absence of the EZ and IZ, marked thinning of the outer retina, and intermittent choroidal hypertransmission, with an intact RPE band.
- Incomplete outer retinal atrophy (iORA): defined by a continuous ELM and a detectable but interrupted EZ in the setting of noticeable outer retinal thinning, without choroidal hypertransmission and with an intact RPE band (Figure 1).

According to the CAM, the term cRORA can be used for atrophy in eyes with or without macular neovascularization (MNV). In this framework, the term geographic atrophy (GA) is typically reserved for atrophy occurring in the absence of current or prior MNV. Similarly, the term nascent GA has been used to describe early OCT features consistent with iRORA when there are no signs of current or previous MNV [11].

The goal of the CAM program was to develop a practical system that helps clinicians assess and counsel patients with AMD. Identifying the earliest OCT-detectable anatomical changes that predict future atrophy is crucial for timely intervention and may accelerate clinical trials aimed at slowing progression. Detecting initial atrophic changes on OCT may also help identify patients who have not yet developed marked symptoms or whose atrophy is not apparent on other imaging modalities, but who may nevertheless be candidates for interventional studies [10].

## 3. OCT Biomarkers Related to Progression to Advanced Non-Exudative AMD

A growing number of OCT biomarkers (now exceeding 100) have been described in the literature, including retinal and choroidal lesions and their characteristics (morphology and reflectivity). Determining which biomarkers are most informative for predicting AMD progression remains an important goal [12]. Doing so may also support the development of robust automated models—using artificial intelligence (AI)—to estimate an individual patient’s risk of progression. All OCT biomarkers of non-exudative AMD progression described in the text are presented in Table 1 below.

### 3.1. Drusen

Drusen are extracellular lipid-rich deposits that accumulate between the RPE and Bruch’s membrane (BM). They may represent changes related to normal aging or an early manifestation of AMD. They consist mainly of a complex mixture of lipids (fatty substances), proteins and cellular debris, and are subject to the hyalinization process to varying degrees, which influences the varied appearance of drusen and their other characteristic features. Drusen are commonly classified by size (small: <63 µm; medium: 63–125 µm; large: >125 µm) and by appearance/consistency (hard: well-defined borders and spherical shape; soft: less distinct borders and mound-like configuration) [13,14]. In addition to size and appearance, features such as distribution, location, and burden (number/area/volume) may have predictive value for disease progression. For example, central location and increasing drusen number have been associated with a higher risk of early AMD progression [15]. OCT enables the extraction of quantitative drusen metrics (e.g., volume, area, and reflectivity). Several drusen phenotypes with distinct imaging features have also been described, including cuticular drusen, calcified drusen, and reticular pseudodrusen (RPD)/subretinal drusenoid deposits (SDD) [14].

#### 3.1.1. Drusen Size

Drusen size is one of the most important risk features and forms the basis of several AMD classification systems. Large drusen are associated with progression to late-stage AMD, particularly GA [16,17]. Color fundus photography (CFP) has historically been the main tool used to categorize drusen as small, medium, or large based on lesion diameter. OCT has increasingly complemented or replaced CFP for risk assessment because it enables more precise, three-dimensional characterization of drusen, including volumetric measurements [18,19].

##### Drusen Volume

Drusen volume is a well-studied risk factor for progression to GA [20,21,22,23,24,25]. Abdelfattah and colleagues first reported that patients with drusen volume exceeding 0.03 mm^3^ were more than four times as likely to progress to late AMD compared with those with smaller drusen volume [21]. Pasricha et al. reported greater drusen volume in regions that later developed GA, detectable 2–4 years before GA onset [26]. In contrast, Chung et al. did not find a statistically significant association between high drusen volume (defined as ≥0.2 mm^3^ within the central 5 mm) and progression risk. The authors used different cutoff values for volume to be considered high and found that a 5 mm area was a better choice compared to a 3 mm circle, which may underestimate volume. It is also important to emphasize that the study population was limited to an Amish cohort, which may affect generalizability [27].

##### Drusen Height

Larger drusen dimensions—particularly increased drusen height—have been associated with other OCT biomarkers of GA risk, such as disruption of the ELM, EZ, and RPE. Greater drusen height may reflect increased mechanical and metabolic stress on the overlying RPE and photoreceptor cells [28]. Oncel et al. proposed a classification based on apical height and basal width that included a category of very large drusen (apical height 55–208 µm and basal width >209 µm) [18].

##### Drusenoid Pigment Epithelium Detachment (dPED)

**Figure 2:**
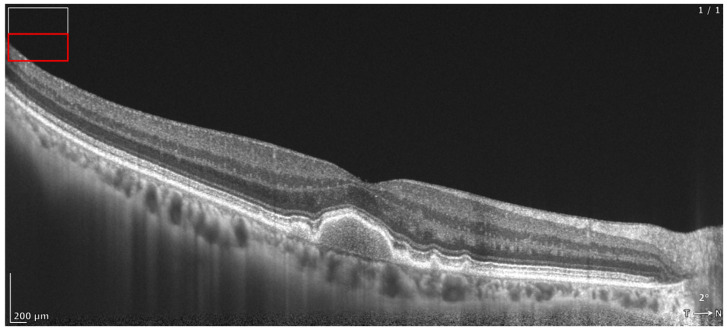
REVO HR OCT B-scan presenting drusenoid pigment epithelium detachment (dPED).

Drusenoid pigment epithelium detachment (dPED) is defined as the separation of the RPE from Bruch’s membrane caused by coalescent large soft drusen. It represents one of the largest drusen-related lesions and is typically defined as reaching at least 350 µm in its narrowest diameter (Figure 2). Drusenoid PED often undergoes subsequent regression/collapse, which may be followed by the development of retinal atrophy. Studies show that approximately 27% to 66% of eyes with drusenoid PED experience collapse or regression over a 3- to 5-year follow-up period [29,30].

Balaratnasingam et al. evaluated the lifecycle of pigment epithelium detachment (PED) by measuring maximal PED volume, height, and diameter to estimate rates of growth and collapse. The rate of drusenoid PED collapse was significantly faster than the rate of PED growth, and larger PEDs collapsed more rapidly [31]. Collapse was often preceded by thickening of the RPE at the druse apex, increased intraretinal hyperreflective foci, acquired vitelliform lesions, and disruption of the RPE–basal lamina–Bruch’s membrane complex (RPE–BL–BrM) [31,32].

Several studies have reported an association between dPED and increased risk of progression to late AMD, particularly GA [16,33]. Yu et al. found that dPED was associated with a significantly increased risk of progression to late AMD and that its presence was also linked to a higher risk of losing ≥15 ETDRS letters from the time of dPED detection, regardless of whether late AMD developed during follow-up [33].

##### RPE–Drusen Complex (RPEDC) Analysis

The RPE–drusen complex (RPEDC) refers to the combined structure of the RPE and the drusen material located between the outer retina and Bruch’s membrane. Quantitative RPEDC parameters reflect drusen burden and can be used for automated drusen quantification [34]. Folgar et al. used semi-automated RPEDC measurements on spectral-domain OCT (SD-OCT). In their approach, the inner RPEDC border was defined by segmentation of the inner surface of the RPE, including the apical surface of drusen, subretinal drusenoid deposits (SDDs), and pigment epithelium detachments (PEDs). The outer RPEDC border was defined by segmentation of the inner surface of Bruch’s membrane [35].

- **RPEDC volume.**

Some SD-OCT platforms include automated quantitative outputs that allow estimation of RPEDC volume. A report from the MACUSTAR study used a deep learning-based algorithm to quantify RPEDC volume and demonstrated a topographic association between large drusen and intraretinal hyperreflective foci, as well as iRORA, in patients with intermediate AMD [36].

- **RPEDC abnormal thinning (RAT).**

Folgar et al. reported that greater RAT volume was associated with progression to GA [35,37]. Abnormal thinning of the RPE is considered an early change in eyes destined to develop GA. Pasricha et al. evaluated the timing of several OCT biomarkers preceding GA onset and found that RAT volume was higher in control regions than in regions that later developed GA across all precursor years, suggesting that abnormal thinning may represent a more generalized process in GA-destined eyes rather than a strictly localized change. During the incident year of GA, RAT volume was significantly higher in GA regions than in control regions [26].

- **RPEDC abnormal thickening.**

In the study by Folgar et al., RPEDC abnormal thickening was treated as a surrogate for OCT-derived drusen volume [35]. Thiele et al. reported that increased RPEDC thickness was the only retinal layer thickness metric (among the layers assessed) that was significantly associated with the development of advanced non-exudative AMD [38].

#### 3.1.2. Drusen Reflectivity

Drusen may show different levels of internal reflectivity on OCT and are often described as having low, medium, or high reflectivity. They can also be classified by reflectivity homogeneity (homogeneous vs non-homogeneous) [39]. The most common phenotype is a convex, homogeneous druse with medium internal reflectivity and no overlying hyperreflective foci [40]. Drusen with low internal reflectivity and reduced overlying RPE reflectivity have been reported as independent predictors of AMD progression, whereas drusen with high internal reflectivity have been associated with relative stability in some studies [41]. Several groups have described related OCT findings of drusen internal heterogeneity during AMD progression [42,43,44]. In practice, the terms OCT-reflective drusen substructures (ODSs), heterogeneous internal reflectivity within drusen (HIRD), and hyporeflective drusen cores (hDCs) partially overlap in how they capture intradrusen reflectivity variation; however, these labels are not always used interchangeably across studies, and careful attention to each study’s definitions is warranted.

##### OCT-Reflective Drusen Substructures (ODSs)

In the AREDS2 Ancillary SD-OCT study, investigators described phenotypic patterns of drusen heterogeneity, termed OCT-reflective drusen substructures (ODSs). Four ODS subtypes were proposed: H-type (high-reflective core), L-type (low-reflective core), C-type (conical debris), and S-type (split druse). H-type consists of a focal, well-circumscribed subvolume of distinct high reflectivity within a druse. L-type consists of a focal, well-circumscribed subvolume of distinct low reflectivity within a druse. C-type corresponds to a conical-shaped druse containing at least three focal, well-circumscribed hyperreflective cores. S-type refers to a druse split into two subvolumes (in any proportion) with distinct levels of reflectivity (low and high). ODSs were reported in approximately 24% of eyes with intermediate AMD and may evolve (transforming between subtypes) before disappearing with drusen regression. ODSs have been proposed as OCT-based biomarkers associated with progression to GA in eyes with intermediate AMD [42].

##### Heterogeneous Internal Reflectivity Within Drusen (HIRD)

Heterogeneous internal reflectivity within drusen (HIRD) has been suggested to indicate increased risk of progression to advanced AMD. In the study by Tan et al., nanoanalytical methods suggested that some OCT features described as HIRD correspond to intradrusen calcified material. Specifically, hyporeflective cores were associated with multilobular nodules composed of hydroxyapatite. In contrast to small hydroxyapatite spherules, which may be reflective on OCT, larger nodules may appear relatively nonreflective and were referred to as “calcified nodules” [43]. Understanding the composition of macular calcifications may help guide future therapeutic strategies and outcome measures in AMD.

##### Hyporeflective Drusen Cores (hDCs)

Hyporeflective drusen cores (hDCs) have been reported to be associated with increased risk of progression to atrophic AMD [12,22,23,44]. He et al. categorized hDCs into three phenotypes: (1) mineralized/calcified and calcifying drusen; (2) cuticular drusen (with or without hypertransmission tails); and (3) degenerating/softening drusen. Among these, the calcified drusen phenotype was most strongly associated with progression risk [44].

##### Hyperreflective Crystalline Deposits (HCDs)

**Figure 3:**
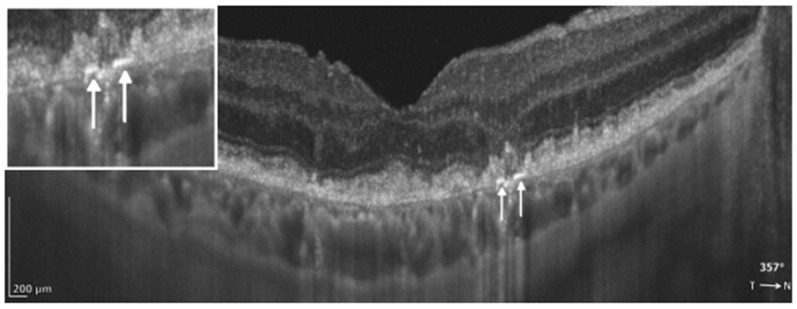
REVO HR OCT B-scan presenting hyperreflective crystalline deposits (HCDs) (white arrows) parallel to Bruch’s membrane, with atrophy of RPE above.

Hyperreflective crystalline deposits (HCDs) are thought to reflect accumulation of cholesterol crystals and may be visible on OCT B-scans as sub-RPE hyperreflective lines parallel to Bruch’s membrane (Figure 3). HCDs have been associated with outer retinal and RPE atrophy (cRORA) [45,46]. Fragiotta et al. described OCT artifacts related to strong reflections from crystalline structures: these appeared as linear artifacts on swept-source OCT (SS-OCT) and as planar artifacts on spectral-domain OCT (SD-OCT). The presence and appearance of these artifacts may relate to cholesterol concentration and the integrity of the RPE [47].

#### 3.1.3. Drusen Appearance

Historically, drusen were classified based on their appearance on color fundus photography (CFP). With multimodal imaging, additional drusen subtypes can be distinguished using characteristic features across imaging modalities. En face OCT has recently been shown to help differentiate cuticular, calcified/refractile, and soft drusen. On en face OCT RPE-projection slabs, these lesions may demonstrate a “donut-shaped” pattern (a round lesion with a hyporeflective core and a hyperreflective rim). Assessment of corresponding choroidal en face projections and confirmation on B-scans can help distinguish among these subtypes [48].

##### Cuticular Drusen

**Figure 4:**
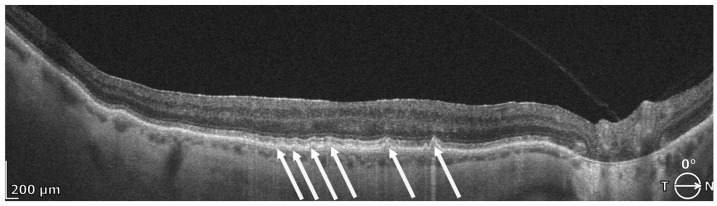
REVO HR OCT B-scan presenting cuticular drusen (white arrows).

Cuticular drusen are numerous small yellow-white deposits that accumulate between Bruch’s membrane and the RPE. On OCT, they typically appear as triangular elevations with steep sides, producing a characteristic “sawtooth” configuration (Figure 4). On fluorescein angiography (FA), they have been described as showing a “Milky Way” or “starry sky” pattern [13,49,50]. Histologically, cuticular drusen contain a homogeneous hyaline core that transmits light efficiently [51]. On en face OCT, choroidal-slab projections may show a “reverse-donut” appearance (a hyperreflective core with a hyporeflective rim), attributed to increased light transmission to the choroid in and around these lesions [48].

Several longitudinal studies have reported that cuticular drusen are associated with the risk of progression to GA [49,52]. Sakurada et al. estimated a 5-year cumulative risk of 28.4% for GA and 8.7% for MNV in eyes with cuticular drusen and suggested that this phenotype may have distinct genetic associations [52]. In contrast, Goh et al. and Chung et al. did not find a statistically significant association between cuticular drusen and progression to late AMD over a shorter follow-up period (2–3 years). Notably, in the population-based Amish cohort studied by Chung et al., cuticular drusen were highly prevalent [27,53].

##### Calcified/Refractile Drusen

**Figure 5:**
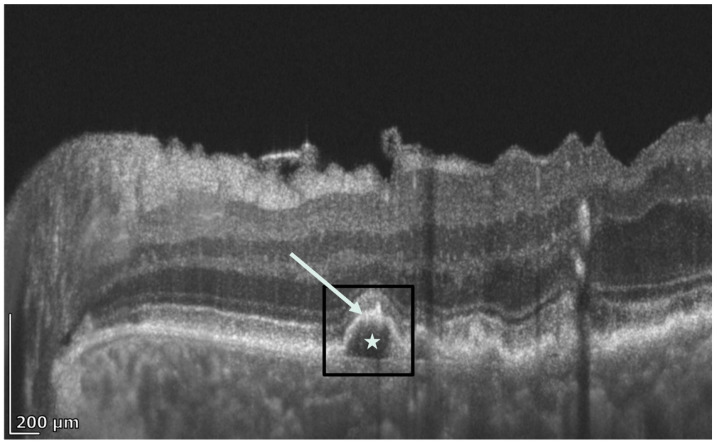
REVO HR OCT B-scan presenting a calcified druse (in black rectangle) with a hiporeflective drusen core (hDC) (white star) and hyperreflective cap (white arrow).

Calcified (refractile) drusen are defined on CFP as drusen with yellowish-white, chalky-white, glistening material beneath the retina [54]. On OCT B-scans, these lesions may correspond to drusen with a hyperreflective cap and hyperreflective spherules, often with associated hyporeflective drusen cores (hDCs) and/or heterogeneous internal reflectivity within drusen (HIRD) (Figure 5) [43].

Liu et al. reported that calcified drusen can appear as dark focal lesions (choroidal hypotransmission defects; hypoTDs) on choroidal en face swept-source OCT (SS-OCT) images. Corresponding B-scans showed drusen with hyporeflective cores and hyperreflective caps. Over time, choroidal hypertransmission defects (hyperTDs) could develop around the edges of hypoTDs, creating a doughnut-like appearance on en face SS-OCT. These doughnut-shaped changes were associated with retinal thinning and met criteria for persistent hyperTD, consistent with cRORA [55]. Similarly, Voichansky et al. described dark choroidal slab lesions corresponding to calcified drusen, attributed to attenuation of light transmission to the choroid [48].

The Classification of Atrophy Meetings (CAM) group recognized calcified drusen as clinically significant lesions that are strongly associated with subsequent development of GA (i.e., complete RPE and outer retinal atrophy; cRORA) [10,55,56].

##### Soft Drusen

**Figure 6:**
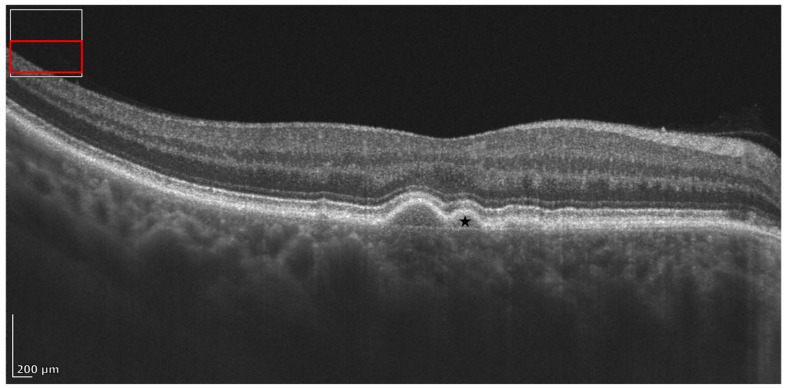
REVO HR OCT B-scan presenting soft confluent drusen (black star).

Soft drusen are confluent yellow-white deposits with indistinct (blurred) borders on CFP. On OCT B-scans, they typically appear as moderately reflective, mound-like elevations of the RPE (Figure 6). On choroidal-slab en face OCT, soft drusen may show isoreflectivity or mild hyporeflectivity [48,56]. Soft drusen are often large with sloping sides, and their contents are enriched in lipids and lipoprotein-derived material. They are associated with increased risk of progression to advanced AMD [57].

### 3.2. Subretinal Deposits

#### 3.2.1. Subretinal Drusenoid Deposits (SDDs)/Reticular Pseudodrusen (RPD)

**Figure 7:**
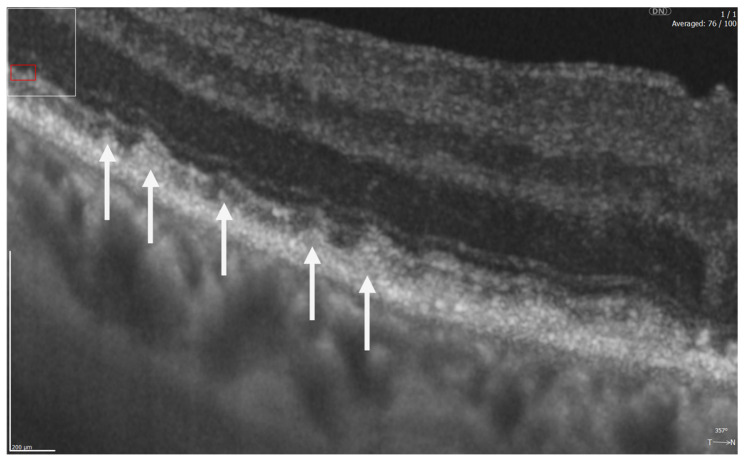
REVO HR OCT B-scan presenting subretinal drusenoid deposits (SDDs) (white arrows).

Subretinal drusenoid deposits (SDDs), also referred to as reticular pseudodrusen (RPD), are deposits located above the RPE in the subretinal space. Clinically, they may appear as well-defined, round or triangular yellowish lesions; on OCT, they correspond to hyperreflective material situated between the photoreceptors and the RPE (Figure 7). On fundus autofluorescence (FAF), SDDs/RPD commonly demonstrate a reticular pattern of decreased autofluorescence [56,58]. On en face OCT, an outer retina projection slab can show multifocal discrete hyperreflective nodules (typical of dot-type SDDs) and a hyperreflective interlaced vermiform pattern characteristic of ribbon (confluent) SDDs [48].

The presence of SDDs/RPD is associated with an increased risk of progression to late AMD, including GA, and prevalence increases with age [12,24,36,59,60,61]. Zhou and colleagues performed a post hoc analysis of the Comparison of AMD Treatments Trials (CATT) and assessed the presence and type of pseudodrusen (dot, reticular, or confluent/ribbon-like) using CFP. They identified RPD as an independent risk factor for AMD progression; dot-type RPD were more strongly linked with the development of MNV, whereas ribbon-like RPD were more strongly linked with the development of GA [60]. Using OCT, Hirabayashi et al. demonstrated that SDDs are associated with the development of cRORA within 2 years [23]. In the study by Trinh et al., greater paracentral photoreceptor thinning in eyes with RPD suggested an association with more advanced retinal degeneration [62]. Mauschitz et al. reported a potential hereditary component of SDDs in first-degree relatives [63]. In addition, several studies have reported that RPD is associated with higher genetic risk scores and with a genome-wide significant association at the ARMS2–HTRA1 locus [64,65].

#### 3.2.2. Acquired Vitelliform Lesion (AVL)

**Figure 8:**
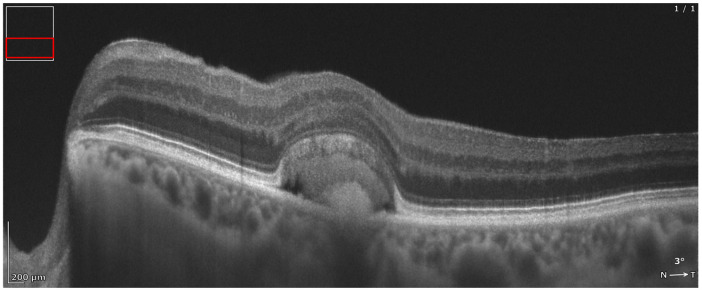
REVO HR OCT B-scan presenting an acquired vitelliform lesion (AVL).

An acquired vitelliform lesion (AVL) appears on color fundus photography as a round, yellow subretinal deposit and typically presents on OCT as a dome-shaped, homogeneous hyperreflective (or hyperreflective/hyperautofluorescent on multimodal imaging) subretinal material (Figure 8). It may be accompanied by a small amount of hyporeflective subretinal fluid, potentially related to incomplete clearance (phagocytosis) of shed photoreceptor outer segments by the RPE. The natural history of AVLs is often characterized by a growth phase followed by partial or complete resorption [50,66]. Histologic studies suggest that AVL material is composed of RPE organelles (3–22% of volume), outer segments (2–10%), lipid droplets (0.2–12%), and unidentified flocculent material (57–59%) [67,68].

Chung et al. demonstrated that the presence of an AVL was independently associated with progression to late AMD over 2 years [27]. In addition, the presence of SDDs and greater AVL height were associated with an increased risk of developing atrophy at the lesion location within 2 years of follow-up [69].

### 3.3. Intraretinal Lesions

#### 3.3.1. Intraretinal Hyperreflective Foci (IHRF)

**Figure 9:**
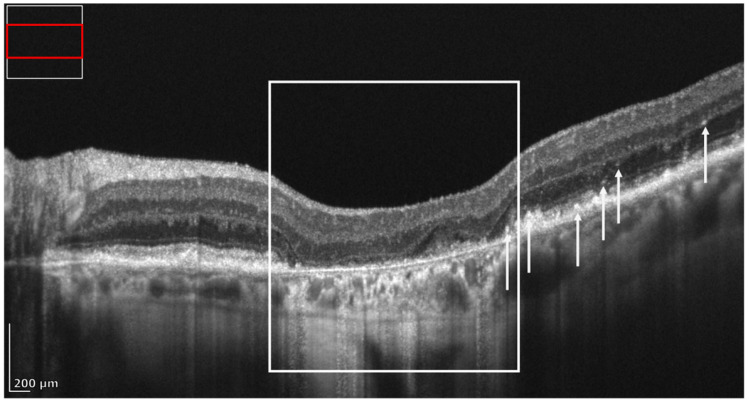
REVO HR OCT B-scan presenting intraretinal hyperreflective foci (iHRF) (white arrows) and cRORA (in the white rectangle).

Hyperreflective foci (HRF) are discrete, round lesions with well-defined borders and high OCT reflectivity, often similar to that of the RPE (Figure 9). They may be located adjacent to the edge or apex of drusen and can be seen within the inner neurosensory retina. HRF are thought to represent, depending on their depth, extracellular pigment granules and outer segment debris (outer HRF) and/or aggregates of migrated or degenerated RPE cells and microglia (inner HRF). Curcio et al. hypothesized that two cellular populations contribute to HRF: anteriorly migrating RPE and posteriorly migrating microglia [70]. Several RPE histologic phenotypes have been linked to hyperreflective structures on OCT B-scans. One example is the “RPE plume,” a comma-shaped HRF configuration that likely corresponds to clusters of migrating RPE cells within the Henle fiber layer [32,70].

HRF are among the most frequently reported OCT predictors of progression to advanced AMD, particularly GA [12,22,23,24,37,44,56,71]. Nassisi et al. quantified HRF volume in eyes with intermediate AMD in a predominantly Caucasian cohort and found that greater HRF volume correlated with a 1-year risk of progression to atrophy, but not to MNV [71]. In contrast, in an Asian population-based study by Kikushima et al., HRF were strongly associated with MNV but not with atrophy; one possible explanation is that advanced atrophic AMD is less prevalent in many Asian cohorts [72]. Schmidt-Erfurth et al. reported different patterns of change preceding late AMD, with features leading to choroidal neovascularization being more drusen-centric, whereas GA-related markers were more strongly associated with neurosensory retinal alterations [73]. In the Amish Eye Study analyzed by Chung et al., the presence of IHRF showed borderline statistical significance; however, limited ethnic diversity may reduce generalizability to other populations [27].

Beyond presence alone, the extent, distribution, and progression of IHRF appear to influence the risk of progression to late AMD. IHRF is most prevalent in the outer retinal layers, and outer-layer IHRF may be associated with the greatest risk of progression [74]. Waldstein et al. reported that eyes with imminent onset of GA did not exhibit drusen or HRF at the foveal center; instead, these features were more often observed at 0.5 mm eccentricity (i.e., near the foveal edge). In contrast, eyes progressing to MNV tended to show accumulation of lesions closer to the center of the macula [75].

Several studies have reported correlations between structural changes such as HRF and functional abnormalities, including prolonged dark adaptation, local reductions in visual sensitivity, and deterioration in visual acuity [76,77]. Hyperreflective specks (HRSs), described by Echols et al., are similar to HRF but smaller in diameter, with lower reflectivity, and are typically localized to the Henle fiber layer and ONL on OCT. These lesions were proposed to represent lipofuscin granules translocating inward within cone photoreceptors [76].

#### 3.3.2. Drusen Ooze

**Figure 10:**
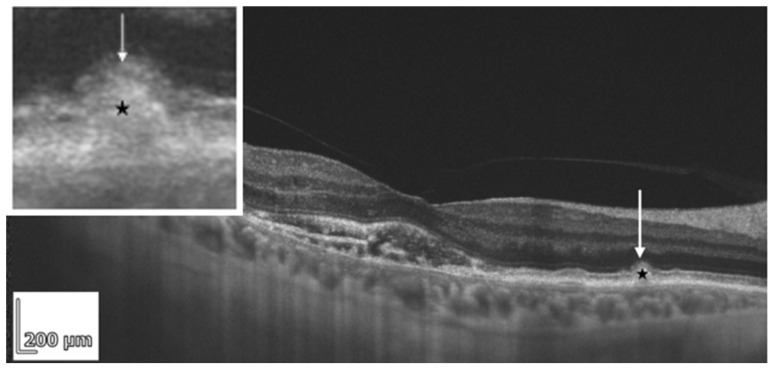
REVO HR OCT B-scan presenting drusen ooze (white arrow) and a soft druse (black star).

Drusen ooze has been described on OCT as small, isoreflective punctate foci within the outer nuclear layer (ONL) and outer plexiform layer (OPL), typically in association with focal RPE defects. The reflectivity of these foci is higher than that of the ONL but lower than the hyperreflective RPE band (Figure 10). It has been hypothesized that these changes represent debris from soft drusen migrating through small RPE defects into the subretinal space shortly before drusen collapse [78].

The presence of drusen ooze has been reported to be a strong risk factor for the development of atrophy within the subsequent 2 years [78]. It has also been described as a predictive sign for progression to iRORA and cRORA over time [79,80].

### 3.4. RPE–BL–BrM Complex Abnormalities

The term RPE–BL–BrM complex was proposed to reflect the presence of additional layers and a potential sub-RPE–BL space that can form in AMD. This space may contain drusenoid material or type 1 macular neovascularization (MNV1) [81,82]. High-resolution histology and transmission electron microscopy (TEM) have identified two major basal deposits: basal laminar deposit (BLamD) and basal linear deposit (BLinD). BLamD accumulates internally to the RPE basal lamina, whereas BLinD is located between the RPE basement membrane and the inner collagenous layer of Bruch’s membrane. When BLamD becomes thick and continuous, it may impede transport between the RPE and the underlying choriocapillaris (ChC), potentially contributing to the formation of soft drusen, BLinD, or both [9,81].

#### 3.4.1. Basal Laminar Deposit (BLamD)/Thin Double-Layer Sign (DLS)

A BLamD is a thickened extracellular matrix located between the RPE plasma membrane and the basal lamina, replacing or incorporating basal RPE infoldings. These deposits are composed primarily of basement membrane proteins and elongated collagen structures [81].

**Figure 11:**
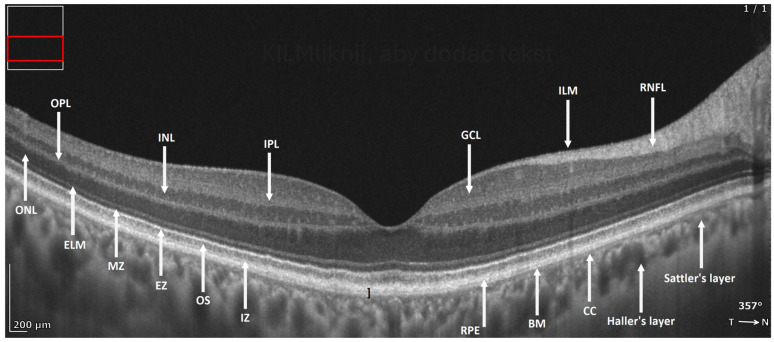
REVO HR OCT B-scan presenting a basal laminar deposit (BlamD) (black buckle) and the laminar structure of the retina and choroid: ILM—inner limiting membrane, RNFL—retinal nerve fiber layer, GCL—ganglion cell layer, IPL—inner plexiform layer, INL—inner nuclear layer, OPL—outer plexiform layer, ONL—outer nuclear layer, ELM—external limiting membrane, MZ—myoid zone, EZ—ellipsoid zone, OS—outer segment, IZ—interdigitation zone, RPE—retinal pigment epithelium, BM—Bruchs’s membrane, CC—choriocapillaris, Sattler’s layer and Haller’s layer.

Chen et al. performed a prospective cross-sectional study using ultrahigh-resolution spectral-domain OCT (UHR SD-OCT) to visualize changes in the RPE–BL–BrM complex [9]. On conventional commercial OCT, this complex typically appears as a single hyperreflective band. With higher-resolution OCT, it can be resolved into three bands (a hyporeflective split between two hyperreflective bands), a feature that is often visible in young healthy eyes and in some eyes with early AMD (Figure 11). The visibility of this split decreases with age. In AMD, the RPE–BL–BrM split has been interpreted as corresponding to the accumulation of a BLamD. Accordingly, measurements of RPE–BL–BrM complex thickness may serve as a surrogate marker of BLamDs in older eyes [9]. Although OCT cannot reliably distinguish BLamDs from BLinDs, a BLamD is generally thicker than a BLinD and therefore is likely to dominate measurements of total complex thickness [81,83].

The “double-layer sign” (DLS) refers to a low-lying elevation of the RPE that can be observed on structural OCT B-scans. This OCT feature typically shows a hyperreflective RPE band separated from an underlying thin hyperreflective band representing Bruch’s membrane [81]. Hirabayashi et al. classified DLS lesions into two types based on thickness. Compared with thin DLSs, thick DLSs contain more than one layer of low-to-medium reflectivity between the RPE and Bruch’s membrane. A thin DLS is thought to correlate with BLamDs, whereas a thick DLS is more suggestive of subclinical MNV [23,84]. Hirabayashi et al. also reported that thin DLSs may be an OCT biomarker of cRORA development within 2 years [23]. Sivaprasad et al. summarized that vascularized (thick) DLSs may be associated with a lower risk of atrophy but can progress to exudative AMD, whereas nonvascularized (thin) DLSs are associated with progression to atrophic AMD [85].

BLamDs may be an indicator of AMD severity. Co-occurrence of BLamDs and SDDs may have synergistic effects on the outer retina and RPE, potentially accelerating progression to atrophy [86].

#### 3.4.2. Basal Linear Deposit (BLinD)

Soft drusen and BLinDs are two forms of similar extracellular lipid-rich material that accumulate on or within Bruch’s membrane. Soft drusen are focal, relatively large deposits with sloping sides and a dome-shaped appearance on OCT, whereas BLinDs are thin, diffusely distributed, and typically not visible clinically, giving the impression of a thickened Bruch’s membrane. Soft drusen may be continuous with adjacent BLinDs, contributing to the “soft” appearance on fundus examination [57].

A BLinD may represent an age-related deposit rather than an AMD-specific lesion. Its accumulation has been associated with increased Bruch’s membrane hyalinization and microscopic RPE abnormalities, suggesting relationships with altered RPE metabolism and/or impaired transport across Bruch’s membrane [87].

### 3.5. Photoreceptor (PR) Layer Abnormalities

Photoreceptors and their connections with neighboring retinal cells appear on OCT B-scans as multiple bands of alternating reflectivity: the outer plexiform layer (OPL; synaptic terminals), Henle fiber layer (HFL; axonal fibers), outer nuclear layer (ONL; photoreceptor nuclei), external limiting membrane (ELM; junctions with Müller glia), ellipsoid zone (EZ; inner segment ellipsoids), outer segments (OSs), and the interdigitation zone (IZ; interface between photoreceptor outer segments and apical RPE processes). Analysis of the continuity and thickness of these bands enables in vivo assessment of photoreceptor integrity and provides important biomarkers for progression to atrophy [11,88]. Goerdt et al. proposed an updated, histology-derived nomenclature in which the OS and IZ region can be subdivided into two subcomponents (OSIZ-1 and OSIZ-2), which may have differential susceptibility to aging and disease [88].

#### 3.5.1. Photoreceptor (PR)/Photoreceptor Segment Layer (PSL) Thinning

Some studies define the photoreceptor (PR) layer as the region between the inner border of the ellipsoid zone (EZ) and the inner border of the RPE, thereby excluding the OPL and ONL [89,90]. Using this definition, Riedl et al. reported that PR thinning was the earliest imaging marker of atrophy, detectable approximately 12 months before OPL subsidence [89]. Brandl et al. showed, in a cross-sectional analysis, that photoreceptor layer and ONL thinning can already be present in early AMD compared with eyes without AMD [90]. In the population-based ALIENOR cohort, Larsen et al. reported that a higher AMD polygenic risk score (PRS) was associated with a thinner photoreceptor segment layer (PSL) at baseline and with longitudinal ONL thinning [91].

Other studies define photoreceptor zone thinning as reduced thickness or volume of the region between the inner portion of the OPL and the inner border of the RPEDC. Using this approach, Pasricha et al. reported that photoreceptor zone thinning and IZ loss were among the earliest changes, detectable up to 4 years before the onset of GA [26].

#### 3.5.2. Outer Nuclear Layer (ONL) Thinning

The ONL is a hyporeflective OCT band representing photoreceptor nuclei. High-resolution OCT can enable visualization of the ONL at cellular and subcellular levels, with photoreceptor nuclei appearing as hyporeflective dots [92]. A key challenge in ONL analysis is separating the Henle fiber layer (HFL) from the ONL; therefore, many automated algorithms segment HFL together with ONL, which can overestimate ONL thickness relative to histologic definitions [93]. Decreased ONL thickness reflects photoreceptor degeneration and is observed with normal aging as well as across stages of AMD [94,95]. Riedl et al. reported that ONL thinning was detectable approximately 18 months before OPL subsidence and continued until subsidence occurred [89]. ONL thinning also correlates with visual function; reduced ONL thickness is associated with decreased retinal sensitivity on microperimetry [96].

#### 3.5.3. Outer Plexiform Layer (OPL) Subsidence

The OPL is a hyperreflective layer formed by synaptic connections between photoreceptors and their post-synaptic cells (bipolar and horizontal cells) [97]. Aresta et al. used a deep learning-based method for automated detection of OPL subsidence on OCT and showed that this feature is a high-risk early biomarker for progression to GA [98]. Ferrara et al. similarly reported that OPL subsidence and inner nuclear layer (INL) subsidence were significantly associated with progression to GA [99].

#### 3.5.4. Hyperreflective Outer Retinal Band (HORB) Abnormalities

Hyperreflective outer retinal bands (HORBs) is a collective term encompassing four OCT bands: the external limiting membrane (ELM), ellipsoid zone (EZ), interdigitation zone (IZ), and retinal pigment epithelium (RPE). Cheung et al. reported that HORB length can be assessed reliably on OCT B-scans using magnitude estimation and that HORB length estimates were significantly associated with age and visual acuity (VA), but not with AMD, in their cross-sectional analysis [100]. Nevertheless, multiple studies have reported that disruption of individual outer retinal bands—particularly the EZ, IZ, and ELM—is associated with progression to advanced AMD and retinal atrophy.

##### Ellipsoid Zone (EZ) Abnormalities

The EZ is a hyperreflective OCT band that reflects the ellipsoid portion of the photoreceptor inner segments. A comprehensive review by Vidal-Oliver et al. describes how assessment of OCT biomarkers—particularly the EZ—has evolved from manual, subjective grading to automated software-based approaches [93].

- **EZ thickness or integrity abnormalities (disruption/attenuation/irregularity/loss/thinning).**

EZ disruption has been associated with a higher risk of progression to advanced AMD overall, as well as to neovascular AMD and GA when analyzed separately [99,101]. Pasricha et al. observed EZ changes up to 3 years before GA onset [26]. Amarasekera et al. recognized EZ changes as predictors of conversion to iRORA and cRORA [80]. Other reports evaluating relationships between drusen and atrophy biomarkers have found an association between EZ disruption and greater drusen height [28]. Flores et al. reported that the combination of iRORA and EZ disruption was associated with a high risk of progression to cRORA within 2 years [101]. Emerging work has also suggested associations between specific plasma metabolites and EZ disruption [102].

Microperimetry testing, used for functional assessment of the retina, demonstrated decreased differential light sensitivity in patients with early AMD with EZ disruption despite good visual acuity results [103]. Lains et al. examined the relationship between dark adaptation (DA) and OCT-based structural changes in AMD and showed that the prevalence of EZ changes and SDDs seems to be associated with impaired dark adaptation [104].

The systematic review and meta-analysis by Trinh et al. showed that EZ abnormality is associated with an increased risk of neovascularization (more so than geographic atrophy) [12]. The authors noted that several studies have highlighted “re-emergence” of EZ integrity after treatment for neovascular AMD [105,106]. This illustrates the potential prognostic utility of OCT biomarkers such as EZ abnormality, which may support earlier treatment initiation and assessment of treatment response.

Recently, the Food and Drug Administration has accepted the rate of change in the area of total EZ loss on OCT scans as a primary outcome measure in a phase III randomized trial of subcutaneous elamipretide in patients with noncentral GA [107].

More and more studies are using automated methods for ellipsoid zone (EZ) loss quantification. The LEAD study observed that EZ loss showed the highest performance in detecting longitudinal changes during evaluation of subthreshold nanosecond laser effects in iAMD [108]. Similarly, Schmidt-Erfurth et al. observed that using pegcetacoplan causes a greater reduction in EZ loss than RPE loss in GA [109].

- **EZ reflectivity (EZR) abnormalities.**

Ellipsoid zone reflectivity (EZR) is thought to relate to the high mitochondrial density in the ellipsoid portion of photoreceptor inner segments and, therefore, to photoreceptor metabolic activity [110]. In contrast to EZ thickness, EZR is not typically provided by standard commercial OCT platforms and generally requires custom post-processing [93]. Thiele and colleagues introduced the term relative EZ reflectivity (rEZR), defined as the ratio of EZ reflectivity to external limiting membrane (ELM) reflectivity [111]. Another approach, normalized EZR, uses en face reconstructions segmented at the EZ plane to quantify EZ reflectivity [112].

Toprak et al. assessed the reflectivity of outer retinal bands using OCT image analysis and found that absolute EZ reflectivity was significantly lower in patients with early non-neovascular AMD compared with healthy controls [113]. Gin et al. reported that generalized reduction in EZ intensity was significantly associated with an intermediate AMD phenotype characterized by macular hyperpigmentation in association with large drusen [114]. Studies using automated approaches have also reported correlations between reduced rEZR and AMD severity, as well as associations with the presence of RPD in subjects with intermediate AMD [115,116]. Liermann et al. demonstrated that lower rEZR was associated with reduced contrast and sensitivity, suggesting potential utility for detecting early photoreceptor dysfunction before overt structural thinning occurs [117].

##### Interdigitation Zone (IZ) Abnormality

The interdigitation zone (IZ) is a hyperreflective OCT band located between the ellipsoid zone (EZ) and the RPE. Anatomically, it reflects the interface between the distal tips of photoreceptor outer segments and the apical processes of the RPE. Using ultrahigh-resolution OCT, Goerdt et al. proposed subdividing the conventional single IZ band into two substructures: OSIZ-1 and OSIZ-2. OSIZ-1 represents a relatively hyporeflective band corresponding to the portion of photoreceptor outer segments not ensheathed by RPE apical processes. In contrast, OSIZ-2 represents a more hyperreflective band corresponding to the outer segment tips in contact with (and interdigitating with) the apical RPE processes. These subcomponents may have different susceptibility to aging and disease [88]. IZ integrity is increasingly recognized as a potential early biomarker of degeneration at the photoreceptor–RPE interface; however, further advances in high-resolution imaging are needed to reliably and consistently delineate the IZ in routine clinical scans [93,118].

IZ loss may be detectable very early in eyes that later develop GA. Pasricha et al. reported IZ loss up to 4 years before GA onset [26]. In their systematic review and meta-analysis, Trinh et al. identified IZ abnormality among the OCT biomarkers most strongly associated with high risk of progression to late AMD [12].

##### External Limiting Membrane (ELM) Abnormalities

The external limiting membrane (ELM) is the thinnest and least reflective of the hyperreflective outer retinal bands (HORBs) seen on OCT and anatomically corresponds to junctional complexes between Müller glia and photoreceptors [97]. Amarasekera et al. reported that ELM integrity abnormalities were predictive of conversion to iRORA and cRORA [80]. In their systematic review and meta-analysis, Trinh et al. identified ELM abnormalities as one of the OCT biomarkers most strongly associated with high risk of progression to late AMD [12]. In the LEAD study, ELM thinning correlated with decreased visual sensitivity [108].

#### 3.5.5. Photoreceptor Segment Abnormalities

Beyond changes in the EZ, IZ, and ELM, abnormalities in the photoreceptor segments themselves, particularly the outer segments (OSs), can be detected on OCT as attenuation, fragmentation, or loss of the thin bands between the EZ and RPE, as well as reduced thickness of the photoreceptor segment region. In intermediate AMD, these changes may reflect early dysfunction at the photoreceptor–RPE interface and impaired renewal/clearance of photoreceptor outer segments. In high-resolution imaging frameworks, segment-level changes may also be conceptualized within the OS/IZ region (e.g., OSIZ subcomponents), which may help localize where disruption begins within the outer segment complex [88].

Photoreceptor segment abnormalities may precede frank atrophy and are frequently captured indirectly by related biomarkers such as PR/PSL thinning and IZ loss. Quantitatively, segment abnormalities can be assessed by measuring the thickness or volume of the photoreceptor segment region (depending on the segmentation definition used) and by evaluating continuity or intensity-based metrics of outer retinal bands [93]. Increasing use of automated and deep learning-based segmentation enables more reproducible longitudinal tracking of subtle segment changes, which may support earlier identification of high-risk eyes and provide sensitive structural endpoints for interventional studies.

### 3.6. Nascent Geographic Atrophy (nGA)

Nascent geographic atrophy (nGA) has been described as a set of early OCT changes that may precede the development of GA. The key features include subsidence of the outer plexiform layer (OPL) and inner nuclear layer (INL), and/or the presence of a hyporeflective wedge-shaped band within the Henle fiber layer (HFL). Several studies have shown that nGA predicts subsequent development of GA, and among nGA-specific findings, OPL subsidence appears to confer the highest risk [99,119]. In the CAM framework, definitions of complete and incomplete RPE and outer retinal atrophy (cRORA/iRORA) were introduced to describe atrophy based on outer retinal changes on OCT [10,11]. The iRORA definition was expanded to incorporate nGA-related criteria [11]. Importantly, nGA features are not required to diagnose iRORA, and nGA can be present without meeting iRORA criteria; however, some lesions may satisfy criteria for both iRORA and nGA. Wu et al. reported that nGA was more strongly associated with future GA development than iRORA or large choroidal signal hypertransmission ≥250 µm (LHyperT) [120,121].

### 3.7. Incomplete RPE and Outer Retinal Atrophy (iRORA)

Incomplete RPE and outer retinal atrophy (iRORA) is defined by OCT features that share key characteristics with complete RPE and outer retinal atrophy (cRORA) but do not meet the size thresholds required for cRORA (i.e., the relevant changes are <250 µm) [11]. Incomplete RORA is characterized by choroidal signal hypertransmission associated with RPE attenuation or disruption, together with evidence of overlying photoreceptor degeneration. Signs of photoreceptor degeneration may include disruption of the ellipsoid zone (EZ) and/or external limiting membrane (ELM), outer nuclear layer (ONL) thinning, subsidence of the inner nuclear layer (INL) and outer plexiform layer (OPL), and/or the presence of a hyporeflective wedge-shaped band within the Henle fiber layer (HFL) [11]. The CAM group recommended the use of en face sub-RPE OCT slabs as a screening approach to detect choroidal hypertransmission suggestive of iRORA, followed by confirmation on the corresponding B-scans [11]. Several studies have reported iRORA as an OCT biomarker associated with progression to advanced atrophic stages of AMD [101,120,122]. Progression risk may be further increased when iRORA co-occurs with other OCT-based features, such as intraretinal hyperreflective foci (IHRF), extrafoveal lesion location, drusen ooze, inner choroid flow deficits (IC FD), and reduced microperimetric retinal sensitivity (MPRS) [79,121,123]. Ameln et al., employing adaptive optics scanning light ophthalmoscope (AOSLO)-based microperimetry, demonstrated that iRORA lesions are associated with marked impairment in retinal sensitivity [124]. Incomplete RORA lesions in subjects with intermediate AMD have been reported to occur with varying frequency across cohorts [36,120]. In an analysis by Nittala et al., rates of progression from iRORA to cRORA were used to assess the effect of intravitreal pegcetacoplan in a post hoc analysis of the FILLY randomized clinical trial; the findings suggested a potential role for pegcetacoplan therapy earlier in the AMD course, before development of GA [125].

### 3.8. Hypertransmission Defects (hyperTDs)

Choroidal hypertransmission defects (hyperTDs) are OCT features reflecting increased transmission of signals into the choroid due to attenuation or loss of the RPE and adjacent outer retinal structures. They are commonly assessed using en face OCT, typically on a sub-RPE slab, which facilitates delineation and measurement of lesion area. Persistent hyperTDs with a greatest linear dimension (GLD) >250 µm on en face OCT imaging have been considered a risk factor for progression to GA [126,127]. Liu et al. described a framework for analyzing persistent choroidal hyperTDs as a potential clinical trial endpoint for therapies aimed at slowing progression from intermediate AMD to late AMD [128].

### 3.9. Choroidal Abnormalities

The choroid and choriocapillaris play a central role in supporting the metabolic demands of the outer retina and RPE. In non-exudative AMD, choroidal structural and perfusion abnormalities have been increasingly studied as potential biomarkers of progression. Structural OCT can provide metrics such as choroidal thickness, whereas OCT angiography (OCT-A) enables noninvasive assessment of choriocapillaris perfusion (e.g., quantification of flow deficits) that may precede or accompany the development of outer retinal atrophy.

Several studies have highlighted associations between reduced choriocapillaris perfusion and progression risk in intermediate AMD. Choriocapillaris flow deficits (FDs) on OCT-A have been reported as risk factors for progression in eyes with AMD [22]. Corradetti et al. further reported that inner choroid flow deficits, together with reduced (scotopic) microperimetric sensitivity, predicted progression to nascent geographic atrophy [123]. Among other OCT-A measurements, vascular parameters such as percent vessel area, total vessel length, and lacunar density in deep capillary plexus (DCP) and choriocapillaris (CC) are particularly sensitive to disease stage and progression. This makes them potential biomarkers for risk stratification and disease monitoring. In contrast, early-appearing changes in superficial capillary plexus (SCP) remain relatively stable, suggesting limited utility in assessing progression [129]. These findings support the concept that choroidal perfusion impairment may contribute to, or serve as an early marker of, the cascade leading to outer retinal degeneration and atrophy.

Interpretation of choroidal biomarkers requires attention to imaging quality and confounders. Both choroidal thickness and OCT-A-derived flow metrics can be influenced by segmentation errors, signal attenuation from overlying drusen or RPE changes, and device- and algorithm-dependent processing [130,131]. Accordingly, choroidal biomarkers may be most useful when interpreted alongside established structural OCT biomarkers (e.g., iRORA, nGA, and persistent hypertransmission defects) and when measured using standardized acquisition and analysis protocols.

## 4. Discussion

Age-related macular degeneration (AMD) remains a major public health problem and can lead to irreversible vision loss with a substantial impact on quality of life [132]. A long interval often separates early disease—frequently asymptomatic—from late-stage complications that cause marked impairment of visual function. Therefore, identifying predictive factors at intermediate stages is important for risk stratification and may support tailored clinical management (e.g., follow-up frequency) and selection of patients for inclusion in interventional trials [125,133].

Because a central challenge in AMD management is estimating an individual patient’s risk of progression to advanced disease, we reviewed the current literature on OCT imaging biomarkers associated with AMD progression. We focused on OCT-based predictors because OCT is a noninvasive, patient-friendly, widely available modality that provides high-resolution cross-sectional imaging and enables three-dimensional quantitative assessment of retinal structures in vivo [7,9,88,92]. OCT can detect early atrophic changes before they become clinically apparent or are classified as atrophy on CFP or FAF. In addition, en face OCT supports improved delineation of lesion boundaries and provides complementary perspectives for biomarker assessment [10,48,55,126,127]. OCT is also increasingly important for defining robust structural endpoints that can improve the efficiency of interventional studies. For example, post hoc analyses have evaluated the transition from iRORA to cRORA as a trial endpoint [125,134]. Ellipsoid zone (EZ) integrity has also been used as a primary structural outcome in FDA-accepted trial designs, including a phase III randomized trial of subcutaneous elamipretide in noncentral GA [107,135]. Moreover, persistent choroidal hypertransmission defects have been proposed as potential endpoints in intermediate AMD [128]. Finally, OCT features can be combined into practical grading systems to stratify progression risk in clinical settings. Lei et al. proposed an OCT-based scoring system that assigns one point for each of four features in each eye (reticular pseudodrusen, outer retinal hyperreflective foci, hyporeflective drusen cores, and large central drusen volume >0.03 mm^3^), yielding a maximum score of 8. Eyes in the highest-risk category (scores 7–8) had approximately threefold higher risk of progression to late AMD than eyes with scores of 5–6 [136]. Future risk calculators may further improve performance by integrating OCT biomarkers with genetic, demographic, and functional factors [137].

OCT-based biomarker assessment enables evaluation of both qualitative characteristics (presence/absence of a lesion) and quantitative characteristics (e.g., lesion size, layer thickness, or reflectivity). Importantly, co-occurrence of multiple biomarkers or specific patterns of retinal change may confer higher progression risk. A key example is the CAM framework for OCT-based staging of atrophy (iORA, cORA, iRORA, and cRORA), which is defined by combinations of outer retinal changes [10,11]. Among these entities, iRORA is currently the most consistently reported prognostic marker for progression to atrophy in the literature [101,120,122]. Increasingly, OCT analysis is performed using automated and semi-automated algorithms. Rapid advances in artificial intelligence (AI)—including machine learning and deep learning—are enabling the development of models that automatically detect and quantify biomarkers and estimate progression risk [138,139]. Examples include automated detection and/or quantification of RPD, iRORA, cRORA, drusen metrics, EZ integrity loss, and OPL subsidence [98,140,141,142,143]. Machine learning has also been used to estimate individualized risk of conversion from intermediate to late AMD by combining OCT biomarkers (e.g., HRF, RPD, drusen, outer retinal thickness) with genetic and demographic variables [73].

Although several OCT biomarkers are supported by multiple studies, other potential predictors of AMD progression remain less well defined. In particular, findings regarding thickness changes in inner retinal layers have been inconsistent across studies. The most frequently replicated inner retinal finding reported in early and/or intermediate AMD is thinning of the ganglion cell layer (GCL) and inner plexiform layer (IPL) [144,145,146]. However, other studies have not observed these changes in early or intermediate AMD [90]. Some reports have suggested that GCL loss is more apparent in eyes with established geographic atrophy rather than in earlier disease stages [147,148]. Muftuoglu et al. demonstrated relatively preserved inner retinal layers in the central fovea and parafovea in mild and intermediate dry AMD; with progression toward atrophy, the IPL thinned. The authors suggested that this may reflect trans-synaptic degeneration of ganglion cell dendrites occurring secondary to photoreceptor loss [149]. Similarly, results regarding retinal nerve fiber layer (RNFL) thickness have been mixed. Some studies reported RNFL thinning in early or intermediate AMD [144,145], whereas others did not find statistically significant differences between eyes with GA, fellow eyes with intermediate AMD, and eyes without AMD [90,147]. Conversely, increased macular RNFL volume has also been reported in GA, possibly reflecting pseudoexpansion of the RNFL into areas of ganglion cell loss [146,148]. Finally, Kaufman et al. reported reduced INL and OPL thickness in atrophic AMD [146], a finding not consistently reported in prior studies [147,150]. Given the lack of consistent evidence on inner retinal changes in AMD, further research is needed to clarify the timing, mechanisms, and clinical relevance of these findings.

## 5. Conclusions

Optical coherence tomography (OCT) has become a cornerstone of diagnosis and longitudinal monitoring in age-related macular degeneration (AMD). OCT enables identification of a broad range of imaging biomarkers and supports both qualitative assessment (presence/absence of lesions) and quantitative assessment (e.g., layer thickness, lesion size, and reflectivity). Improved characterization of structural changes across AMD stages contributes to a better understanding of disease pathophysiology and supports the development of therapeutic strategies and imaging-based trial endpoints. Because non-exudative AMD can have a prolonged, often minimally symptomatic phase before advanced complications occur, earlier detection of high-risk structural features is critical for timely risk stratification and follow-up. Recent evidence suggesting that photoreceptor (PR) thinning, outer nuclear layer (ONL) thinning, and interdigitation zone (IZ) disruption can occur early in the course of AMD highlights their potential roles as candidate endpoints and enrichment biomarkers in interventional studies. Finally, emerging artificial intelligence (AI) approaches that automate the detection and quantification of OCT biomarkers may further improve reproducible monitoring and individualized risk assessment.

## 6. Future Directions

Among the growing number of biomarkers, it is important to identify those most significant in assessing the risk of AMD progression. Future analysis should focus on the changes that appear at the earliest stage. PR and ONL thinning, as well as IZ disintegration, seem to be among the first lesions to appear in AMD. Furthermore, some changes have been shown to occur in the inner layers of the retina, but these reports are contradictory, and further research is needed. This review did not find any studies that examined the possible relationship between iORA and cORA, terms defined by the CAM group, and the risk of AMD progression. This topic is perhaps worth considering, as these two definitions have not yet encompassed RPE atrophy and are characterized by the prevalence of atrophy, thinning, or merely disruption of the EZ layer, which has been recognized in some studies to be reversible after therapy. Although OCT-based parameters are the most frequently reported in the literature, standardized protocols for measuring many of these biomarkers are still lacking. Inconsistency in the definitions of OCT-diagnosed lesions contributes to discrepancies in measurements and analyses of different features. Therefore, it is necessary to establish precise definitions and standardize research protocols, enabling consistent interpretation of results and subsequent use of biomarkers in clinical care and future trials.

Searching for new OCT markers and testing existing ones is important due to the increasing development of OCT technology. The increasingly higher resolution implementation in OCT devices, the development of telemedicine, and the creation of home OCT devices for self-imaging are promising. The rapidly developing field of artificial intelligence offers tools that improve monitoring, diagnosis, and risk assessment of AMD progression. It enables automatic segmentation of morphological features, proposes pathophysiological patterns based on identified associations between markers, and helps classify patients according to the risk of AMD progression. The literature contains attempts to predict individual risk of disease progression using state-of-the-art AI methods. This provides increasing opportunities for effective screening and intervention at the earliest possible moment and is aimed at a specific goal.

## Figures and Tables

**Table 1:** List of OCT biomarkers related to progression to advanced non-exudative AMD and their abbreviations.

OCT Biomarkers	
**Drusen**	
	**Drusen size:**
	Drusen volume
	Drusen height
	Drusenoid pigment epithelium detachment (dPED)
	RPE–drusen complex (RPEDC)
	RPEDC volume
	RPEDC abnormal thinning (RAT)
	RPEDC abnormal thickening
	**Drusen reflectivity:**
	OCT-reflective drusen substructures (ODSs)
	Heterogeneous internal reflectivity within drusen (HIRD)
	Hyporeflective drusen cores (hDCs)
	Hyperreflective crystalline deposits (HCDs)
	**Drusen appearance:**
	Cuticular drusen
	Calcified/refractile drusen
	Soft drusen
**Subretinal deposits**	
	Subretinal drusenoid deposits (SDD)/reticular pseudodrusen (RPD)
	Acquired vitelliform lesion (AVL)
**Intraretinal lesions**	
	Intraretinal hyperreflective foci (iHRF)
	Drusen ooze
**RPE–BL–BrM complex abnormalities**	
	Basal laminar deposit (BLamD)/thin double-layer sign (DLS)
	Basal linear deposit (BLinD)
**Photoreceptor layer abnormalities**	
	**Photoreceptor (PR)/photoreceptor segment layer (PSL) thinning**
	**Outer nuclear layer (ONL) thinning**
	**Outer plexiform layer (OPL) subsidence**
	**Hyperreflective outer retinal band (HORB) abnormalities:**
	Ellipsoid zone (EZ) abnormalities:
	EZ thickness or integrity abnormalities (disruption/attenuation/irregularity/loss/thinning)
	EZ reflectivity (EZR) abnormalities
	Interdigitation zone (IZ) abnormality
	External limiting membrane (ELM) abnormality
	**Photoreceptor segment abnormalities:**
	Outer segment (OS)
**Nascent geographic atrophy (nGA)**	
**Incomplete RPE and outer retinal atrophy (iRORA)**	
**Hypertransmission defects (hyperTDs)**	
**Choroidal abnormalities**	
	Choroidal thickness
	OCT-A-derived flow metrics

## Data Availability

No new data were created or analyzed in this study. Data sharing is not applicable to this article.

## Abbreviations

The following abbreviations are used in this manuscript:

AMD	Age-related Macular Degeneration
GA	Geographic Atrophy
OCT	Optical Coherence Tomography
iRORA	Incomplete Retinal Pigment Epithelium and Outer Retinal Atrophy
cRORA	Complete Retinal Pigment Epithelium and Outer Retinal Atrophy
RPE	Retinal Pigment Epithelium
CFP	Color Fundus Photography
FAF	Fundus Autofluorescence
NIR	Near-Infrared
FA	Fluorescein Angiography
ICG-A	Indocyanine Green Angiography
OCT-A	Optical Coherence Tomography Angiography
iAMD	Intermediate Age-Related Macular Degeneration
CAM	Classification of Atrophy Meetings
ONL	Outer Nuclear Layer
ELM	External Limiting Membrane
EZ	Ellipsoid Zone
IZ	Interdigitation Zone
INL	Inner Nuclear Layer
OPL	Outer Plexiform Layer
HFL	Henle Fiber Layer
cORA	Complete Outer Retinal Atrophy
iORA	Incomplete Outer Retinal Atrophy
HR	High-Resolution
MNV	Macular Neovascularization
AI	Artificial Intelligence
BM/BrM	Bruch’s Membrane
RPD	Reticular Pseudodrusen
SDD	Subretinal Drusenoid Deposit
dPED	Drusenoid Pigment Epithelium Detachment
PED	Pigment Epithelium Detachment
RPE-BL-BrM	Retinal Epithelium Pigment–Basal Lamina–Bruch’s Membrane Complex
RPEDC	Retinal Epithelium Pigment–Drusen Complex
SD-OCT	Spectral-Domain Optical Coherence Tomography
RAT	RPEDC Abnormal Thinning
ODS	Optical Coherence Tomography–Reflective Drusen Substructures
HIRD	Heterogeneous Internal Reflectivity within Drusen
hDC	Hyporeflective Drusen Core
HCD	Hyperreflective Crystalline Deposit
SS-OCT	Swept-Source–Optical Coherence Tomography
hypoTDs	Hypotransmission Defects
hyperTDs	Hypertransmission Defects
CATT	Comparison of AMD Treatments Trials
AVL	Acquired Vitelliform Lesion
iHRF	Intraretinal Hyperreflective Foci
HRF	Hyperreflective Foci
TEM	Transmission Electron Microscopy
BLamD	Basal Laminar Deposit
BLinD	Basal Linear Deposit
ChC/CC	Choriocapillaris
DLS	Double-Layer Sign
UHR	Ultrahigh-Resolution
PR	Photoreceptor
OS	Outer Segment
PSL	Photoreceptor Segment Layer
PRS	Polygenic Risk Score
HORB	Hyperreflective Outer Retinal Band
VA	Visual Acuity
DA	Dark Adaptation
EZR	Ellipsoid Zone Reflectivity
rEZR	Relative Ellipsoid Zone Reflectivity
nGA	Nascent Geographic Atrophy
MPRS	Microperimetric Retinal Sensitivity
AOSLO	Adaptive Optics Scanning Light Ophthalmoscope
GLD	Greatest Linear Dimension
FD	Flow Deficit
DCP	Deep Capillary Plexus
SCP	Superficial Capillary Plexus
FDA	Food and Drug Administration
GCL	Ganglion Cell Layer
RNFL	Retinal Nerve Fiber Layer
